# The taming of an impossible child: a standardized all-in approach to the phylogeny of Hymenoptera using public database sequences

**DOI:** 10.1186/1741-7007-9-55

**Published:** 2011-08-18

**Authors:** Ralph S Peters, Benjamin Meyer, Lars Krogmann, Janus Borner, Karen Meusemann, Kai Schütte, Oliver Niehuis, Bernhard Misof

**Affiliations:** 1 Zoologisches Forschungsmuseum Alexander Koenig, Adenauerallee 160, D-53113 Bonn, Germany; 2 Institut für Systemische Neurowissenschaften, Universitätsklinikum Hamburg-Eppendorf, Martinistrasse 52, D-20246 Hamburg, Germany; 3 Staatliches Museum für Naturkunde Stuttgart, Rosenstein 1, D-70191 Stuttgart, Germany; 4 Zoologisches Institut der Universität Hamburg, Martin-Luther-King-Platz 3, D-20146 Hamburg, Germany; 5 Zoologisches Museum Hamburg, Martin-Luther-King-Platz 3, D-20146 Hamburg, Germany

## Abstract

**Background:**

Enormous molecular sequence data have been accumulated over the past several years and are still exponentially growing with the use of faster and cheaper sequencing techniques. There is high and widespread interest in using these data for phylogenetic analyses. However, the amount of data that one can retrieve from public sequence repositories is virtually impossible to tame without dedicated software that automates processes. Here we present a novel bioinformatics pipeline for downloading, formatting, filtering and analyzing public sequence data deposited in GenBank. It combines some well-established programs with numerous newly developed software tools (available at http://software.zfmk.de/).

**Results:**

We used the bioinformatics pipeline to investigate the phylogeny of the megadiverse insect order Hymenoptera (sawflies, bees, wasps and ants) by retrieving and processing more than 120,000 sequences and by selecting subsets under the criteria of compositional homogeneity and defined levels of density and overlap. Tree reconstruction was done with a partitioned maximum likelihood analysis from a supermatrix with more than 80,000 sites and more than 1,100 species. In the inferred tree, consistent with previous studies, "Symphyta" is paraphyletic. Within Apocrita, our analysis suggests a topology of Stephanoidea + (Ichneumonoidea + (Proctotrupomorpha + (Evanioidea + Aculeata))). Despite the huge amount of data, we identified several persistent problems in the Hymenoptera tree. Data coverage is still extremely low, and additional data have to be collected to reliably infer the phylogeny of Hymenoptera.

**Conclusions:**

While we applied our bioinformatics pipeline to Hymenoptera, we designed the approach to be as general as possible. With this pipeline, it is possible to produce phylogenetic trees for any taxonomic group and to monitor new data and tree robustness in a taxon of interest. It therefore has great potential to meet the challenges of the phylogenomic era and to deepen our understanding of the tree of life.

## Background

Reconstructing the phylogeny of organisms, the tree of life, is one of the major goals in biology and is essential for research in other biological disciplines ranging from evolutionary biology and systematics to biological control and conservation. In phylogenetics, molecular characters have become an indispensable tool, since they can be collected in a standardized and automated way. This is indicated by the exponential growth of published data, with a current doubling time of approximately 30 months [1] and expected massively accelerated data generation over the next several years. The sequencing of expressed sequence tags (ESTs), complete genomes and countless single-gene fragments has resulted in enormous, yet highly incomplete and unbalanced, data sets accessible via public databases such as the National Center for Biotechnology Information (NCBI) GenBank, the European Molecular Biology Laboratory (EMBL) and the DNA Database of Japan (DDBJ).

The accumulation of new data is, of course, important, but the potential of the currently available data for phylogenetic analysis has not yet been sufficiently explored. McMahon and Sanderson [2], Sanderson *et al*. [3] and Thomson and Shaffer [4] have published their attempts to use molecular data from public databases and to process them for phylogenetic analysis. However, these approaches, while valuable and trend-setting, did not offer thorough solutions and call for extension, improvements and updates in terms of generalization, detail, analysis and degree of automation. Any new approach must offer solutions to deal with data scarcity, poor data overlap, nonstationary substitution processes, base compositional heterogeneity and data quality deficits. In this study, we address these problems with a newly developed bioinformatics pipeline. We use a large exemplar taxon for which far more than 100,000 sequences have been published and show that comprehensive analyses can potentially deliver new results which were not available from each included data set separately.

As an exemplary taxon, we chose the insect order Hymenoptera, which comprises prominent groups such as bees, ants and wasps, the latter including the overwhelming armada of parasitoid species [5]. The Hymenoptera seem well-suited to demonstrate the power of our approach, since the taxon is megadiverse and offers a number of phylogenetic challenges, including many unresolved relationships and well-known problems that are associated with so-called long-branch taxa and rapid radiations (see, for example, [6-8]). Over a long period, comparatively few authors tried to resolve the phylogenetic relationships of the major lineages of Hymenoptera (see, for example, [9-16]). In recent years, however, interest and effort in solving higher-level relationships within the Hymenoptera have notably increased and led to the publication of an extensive analysis based exclusively on morphological characters [17], a study using complete mitochondrial genomes [18], a supertree approach using previously published trees [19], a phylogenetic estimate based on EST data [20] and a taxon-rich four-gene study [21]. In the past five years, complete nuclear genomes of several Hymenoptera species have been sequenced. Most noteworthy in this context are the genomes of the honey bee *Apis mellifera *[22] and the jewel wasp *Nasonia vitripennis*, with its sibling species *N. giraulti *and *N. longicornis *[23]. These genomes contributed significantly to the amount of sequence data available for Hymenoptera. However, their number is still too small to profitably augment phylogenetic analyses.

Overall, there are only few phylogenetic hypotheses on major lineages within Hymenoptera that are generally accepted. These are as follows: (1) "Symphyta" (sawflies) are paraphyletic, with the absence of the constriction between the first and second abdominal segments (that is, the wasp waist) as a symplesiomorphic character; (2) Apocrita (wasp-waisted wasps) are monophyletic (see, for example, [24]); (3) Xyelidae are sister group to all other Hymenoptera (see, for example, [25-27]); (4) Orussidae are sister group to Apocrita (see, for example, [17,18,27]) and (5) Aculeata (stinging wasps; Apoidea, Chrysidoidea and Vespoidea) are monophyletic (see, for example, [28]). In addition, most of the 22 currently recognized superfamilies are presumed to be monophyletic (see [29] for a synopsis). Numerous relationships within Hymenoptera are still unresolved. Among them, the most intriguing ones are the phylogeny of the major lineages within Apocrita, and in particular what the sister group of Aculeata is, and the monophyly and phylogeny of Proctotrupomorpha *sensu *Rasnitsyn 1988 [13] (Chalcidoidea, Cynipoidea, Diaprioidea, Mymarommatoidea, Platygastroidea and Proctotrupoidea).

In this study, we present a standardized, fast and transparent bioinformatics pipeline to collect, filter and analyze public sequence data deposited in GenBank. The pipeline is designed to be generally applicable in terms of taxa, genes and the variety of potential users. We apply this pipeline to sequences of Hymenoptera and discuss our results against the background of current hypotheses on two selected questions: the phylogeny of the major lineages within Apocrita and the monophyly and phylogeny of Proctotrupomorpha. Additionally, we use the results to diagnose persistent problems in the hymenopteran tree. Finally, we illustrate the merit of being able to easily generate trees from available sequence data at a time when data sets are accumulating at an ever-increasing speed.

## Methods

**Figure 1:**
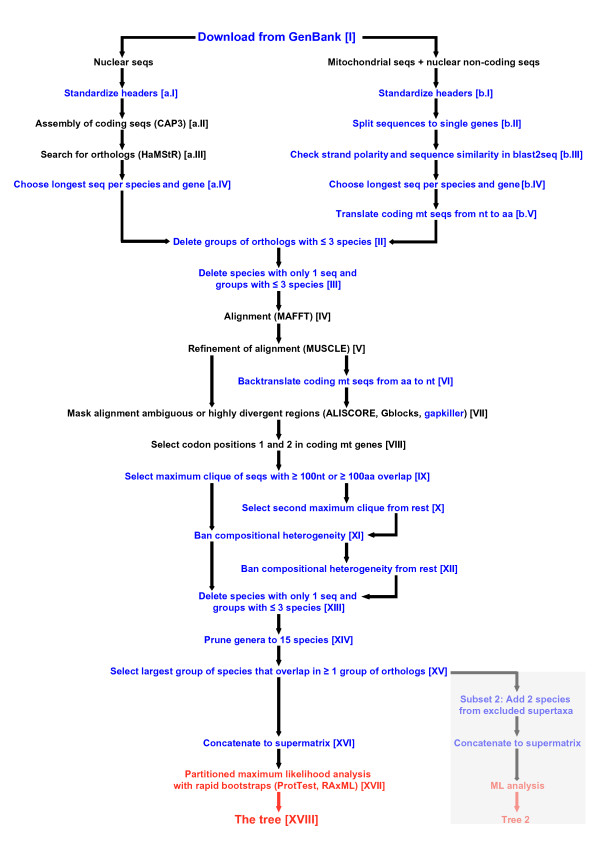
**Outline of our pipeline that processes GenBank sequence data for phylogenetic analysis**. Steps that are executed by newly developed scripts are highlighted in blue, and external programs are written in parentheses after step description. Steps that directly refer to the phylogenetic analysis are highlighted in red. The additional procedure to infer subset 2 is shaded in gray.

We developed a bioinformatics pipeline that includes automated data retrieval, processing, filtering and analysis of sequence data using available programs in combination with newly developed scripts. The individual steps of the pipeline are illustrated in Figure 1. Those steps that are executed by new scripts are highlighted in blue. These scripts can be downloaded from http://software.zfmk.de/ or accessed as part of Additional file 1. They have been written in the Ruby or Perl programming language and will run on any Linux operating system. Each of our scripts comes with a manual that provides more detailed information on what it does and how to use it (manuals are available at http://software.zfmk.de/ and also are located in Additional file 1). Table 1 summarizes all new scripts and their respective tasks. To maneuver through the pipeline, each script has to be manually started with the output from the preceding step. This allows the user to manually interfere at each step or to modify the pipeline to adapt it to new demands. In the following paragraphs, we explain the individual steps of the pipeline using the example of the analysis of Hymenoptera sequences deposited in GenBank.

**Table 1:** New scripts used in our pipeline^a^

Step	Number	Script
Download from GenBank	[I]	*proseqco*
Standardize headers	[a.I], [b.I]	*header_standardizer*
Split sequences to single genes	[b.II]	*multiple_sequence_splitter*
Check strand polarity and sequence similarity	[b.III]	*checking_seq*
Choose longest sequence per species and gene	[a.IV], [b.IV]	*choose_longest_seq*
Translate coding mitochondrial sequences from nucleotides to amino acids	[b.V]	*dna2aa*
Delete groups of orthologs with three or fewer species	[II], [III], [XIII]	*small_groups_deleter*
Delete species with only one sequence	[III], [XIII]	*taxon_deleter*
Backtranslate coding mitochondrial sequences from amino acids to nucleotides	[VI]	*aa2dna*
Mask gappy regions in alignment	[VII]	*gap_killer*
Select maximum clique of overlapping sequences	[IX], [X]	*minimum_sequence_overlap*
Ban compositional heterogeneity	[XI], [XII]	*nucleotide_chi*
Prune genera to best represented species	[XIV]	*prune_genera*
Select largest group of species that overlap in at least one group of orthologs	[XV]	*reduce2leading_gene*
Concatenate alignments	[XVI]	*concatenator*

^a^Available at http://software.zfmk.de/ and in Additional file 1. All scripts were written in Ruby, except for *checking_seq*, which was written in Perl. Numerals (column "Number") correspond to those in Figure 1.

### Sequence data retrieval and data processing

We downloaded all sequences of Hymenoptera deposited in GenBank 172.0 (as of 18 August 2009) with the aid of the script *proseqco *[I] (Roman numerals in square brackets correspond to those in Figure 1). The script searched for the query taxon (Hymenoptera) in the nucleotide and in the EST database of GenBank (NCBI) and stored the sequences of each species in a separate Fasta file. Mitochondrial sequences plus nuclear noncoding sequences (ITS1, ITS2 and nuclear rRNA) (right path b) and all other nuclear sequences (left path a) were retrieved in two separate downloads. For outgroup comparison, we additionally retrieved sequence data of the transcriptome, the nuclear noncoding genes and the complete mitochondrial genome of *Bombyx mori *(Lepidoptera), *Aedes aegypti *(Diptera) and *Tribolium castaneum *(Coleoptera). The gi numbers of all downloaded sequences are listed in Additional file 2.

#### Left path a

The nuclear sequences were assembled into contigs for each species using the sequence assembly program CAP3 [30] [a.II]. Orthologous sequences were identified using HaMStR 1.3 [31] [a.III]. We used the Insecta core set (available at http://www.deep-phylogeny.org/hamstr/download/datasets/hmmer2/) to build hidden Markov models (default settings). The genome of *A. mellifera *was chosen for the reciprocal BLAST search [31]. (If sequences of other taxa are processed, a different core set and a different species for the reciprocal BLAST search will have to be selected.) We chose HaMStR as the currently most practicable tool to automatically assign orthology among nucleotide and EST sequence data. During the HaMStR orthology prediction, all nucleotide sequences are translated into the corresponding amino acid sequences.

#### Right path b

The mitochondrial sequences and the nuclear noncoding sequences deposited in GenBank often include regions that span more than just one gene. In these instances, the script *multiple_sequence_splitter *uses information from the corresponding GenBank file to split sequences into fragments that correspond to single genes; that is, it creates multiple sequence files of single genes [b.II]. This step was serially applied to each file that we obtained from the previous step by means of a shell script. (See the *multiple_sequence_splitter *manual for a description of how to do this. Any step of the pipeline that had to be serially applied to a set of files was executed by means of a similar shell script [a.I, a.IV, b.I, b.II, b.IV, b.V, IV, V, VI, VII, IX, X, XI and XII].) In each of the obtained files, we used the script *checking_seq *to check for consistent strand polarity and overall similarity between sequences [b.III]. This was done to revert sequences with deviating strand polarity, to exclude wrongly annotated sequences and to ensure that all sequences in a single-gene file were orthologous. The script *checking_seq *compares a template of a gene with all the sequences of the single-gene files that were created in step [b.II] in blast2seq [32]. The identity (blast2seq results) between template and target sequence had to be more than 15 nucleotides. Otherwise, the reverse complement of the target sequence was checked, and hits were reverted. If identities were still below the match threshold, the target sequences were compared with a second, third or fourth template. Primary templates were taken from *A. mellifera*. (If sequences of other taxa are processed, other templates will have to be selected.) We randomly selected sequences from previously successfully checked sequences as subsequent templates. A maximum of four templates were used before we finally discarded a sequence. Then, to prepare the remaining sequences for the subsequent alignment, all coding mitochondrial sequences were translated from nucleotide to corresponding amino acid sequences with the aid of the script *dna2aa*, which uses the respective GenBank information for this task [b.V]. Steps b.IV and b.V of our pipeline are automatically consecutively executed when using the script batch1_bIVtobV.sh. (See manual of batch scripts for details.)

#### Both paths

Sequence headers of all sequences were standardized to ">species,family,gi no." with the aid of the script *header_standardizer*, which uses the data included in the GenBank entries [a.I and b.I]. If multiple sequences were available for a given species and gene after respective steps [a.I to a.III] and [b.I to b.III], we chose the longest sequence from the unaligned multiple sequence files [a.IV and b.IV]. This was done by using the script *choose_longest_seq*.

#### Converged paths

We obtained numerous groups of orthologous sequences from path a and path b. Groups of orthologs that comprised three or fewer species were deleted by the script *small_groups_deleter *[II]. To increase data density, we discarded all species with only a single sequence in the data set by using the script *taxon_deleter *and again deleted groups of orthologs with three or fewer species by using *small_groups_deleter *[III].

### Multiple sequence alignment and alignment masking

Orthologous sequences were aligned with MAFFT v6.712b using the auto option [IV]. Depending on the size of an alignment, MAFFT automatically chooses a suitable alignment option, such as L-INS-i for < 200 sequences and FFT-NS-2 for > 2,000 sequences [33,34]. All alignments were subsequently refined with the refinement option in MUSCLE version 3.7 [35] [V]. These are powerful alignment tools that allow processing very large data sets in reasonable time. Steps II through VI of our pipeline are automatically consecutively executed when using the script batch2_IItoVI.sh. (See the manual of batch scripts for details.) Aligned and refined mitochondrial amino acid sequences were then translated back into nucleotide sequences with the aid of the script *aa2dna*, which uses the corresponding reading frame information from the GenBank file [VI]. From this point on, we proceeded with nucleotide sequences for all mitochondrial sequences and nuclear noncoding sequences, as well as with amino acid sequences for the nuclear coding sequences (available since step [a.III]).

Ambiguously aligned or highly diverged regions of the alignment were masked with three different algorithms [VII]. We applied ALISCORE [36,37] and ALICUT [38] for noncoding nucleotide sequences and for nuclear amino acid sequences (default settings). Since the multiple sequence alignment of 28S rRNA was too big to be processed with ALISCORE, we used Gblocks 0.91b [39,40] for 28S instead (block parameter settings: (1) number of included seq/2 = 1020, (2) 1020, (3) 5, (4) 10, and (5) all). Finally, we used the script *gapkiller *to identify and delete sites with more than 70% gaps in coding mitochondrial sequences. Then we masked all third codon positions of mitochondrial coding sequences [VIII] and concatenated all tRNA alignments to one single alignment.

### Species and sequence subset selection

In each group of orthologous sequences, we selected the largest group of species in which the sequences of all species overlap in at least 100 nucleotide or amino acid positions [IX]. This was done with the aid of the script *minimum_sequence_overlap*. The script applies a maximum clique algorithm. Generally, a maximum clique search is a way to find the largest group of items that fulfill a certain pairwise criterion. (See Additional file 3 for a short introduction to maximum cliques.) This approach is the formal solution to guarantee that our overlap criterion is fulfilled. Species that were not included in this first maximum clique were considered again in a search for a second maximum clique using the same criteria and the same script as before [X]. So, for each gene, we retained two separate files with groups of orthologous sequences: the first and the second maximum clique, respectively. Sequences that were not included in either of the maximum cliques were discarded.

To identify sequences that showed compositional heterogeneity in each group of orthologous nucleotide sequences, we used the script *nucleotide_chi*. The script applies a χ^2 ^test (test procedure identical to the χ^2 ^test implemented in TREE-PUZZLE [41]) and proceeds with excluding sequences with a base composition that significantly deviates until all sequences show compositional homogeneity [XI]. Since excluded sequences could comprise another set of homogeneous sequences, they were again tested with the same procedure as before to obtain a second group of sequences with compositional homogeneity [XII]. Sequences that did not end up in either of the two groups with compositional homogeneity were discarded. After discarding numerous sequences in steps IX through XII, we again excluded species with only one sequence in the data set by using the script *taxon_deleter *and groups of orthologs with three or fewer species by using the script *small_groups_deleter *[XIII]. Next, we pruned species-rich genera to the 15 species that were best represented in the data set by using the script *prune_genera*. The representation criteria were, in this order, (1) the number of sequences in the data set and (2) the overall length of the sequence in the data set [XIV].

In a final subset selection step, we ensured that all species to be included in this subset overlap in at least one gene fragment of at least 100 nucleotide or amino acid positions [XV]. With the aid of the script *reduce2leading_gene*, we pruned the data set to those species that were present in the most sequence-rich group of orthologs. This was the largest group of species that fulfilled the overlap criterion. In case of Hymenoptera, this group was a group of COX1 sequences. All corresponding sequences were concatenated with the script *concatenator *to one supermatrix. This supermatrix is referred to as "subset 1" [XVI]. Steps IX through XVI of our pipeline are automatically consecutively executed when using the script batch3_IXtoXVI.sh. (See manual of batch scripts for details.) In addition to subset 1, we selected a second subset. To accomplish this, we made concessions to systematic considerations and added to subset 1 representatives of Hymenoptera families that were excluded by any of the previous filtering steps. If more than two species of the respective families were available, we selected the two best-represented species using criteria identical to those in step [XIV]. With those sequences reincluded in the respective groups of orthologs, the tests for compositional heterogeneity (as described in step [XI]) were repeated and all sequences were finally concatenated to a supermatrix. This supermatrix is referred to as "subset 2."

### Tree reconstruction

Phylogenetic inference of subset 1 and of subset 2 was done under the maximum likelihood (ML) optimality criterion in partitioned analyses with RAxML 7.2.8 [42,43] under the GTRCAT model. Analyses were computed on HPC Linux clusters, 8 nodes with 12 cores each, at the Regionales Rechenzentrum Köln (RRZK) using Cologne High Efficient Operating Platform for Science (CHEOPS); input was done in phylip format; and conversion of Fasta to phylip was done using Readseq [44] [XVII]. Nuclear coding genes were treated as one partition (PROTCAT model, substitution matrix LG + F, taken from ProtTest [45]). All other groups of orthologs were treated as separate partitions (32 partitions in total). (See Additional file 4 for the character partitions of subset 1 and 2.) We applied the rapid bootstrap algorithm [46] with a subsequent tree search. The numbers of bootstrap replicates were estimated on the fly by the "bootstopping" criteria implemented in RAxML 7.2.8 (default settings) [47]. The analyses yielded two trees. These trees are referred to as "tree 1" (corresponding to subset 1) and "tree 2" (corresponding to subset 2). Trees were edited in Dendroscope [48] [XVIII].

### Hymenoptera systematics

We follow the terminology of [29] for supraspecific taxa of Hymenoptera.

## Results

We downloaded 122,723 Hymenoptera sequences from GenBank 172.0 (as of 18 August 2009), including those of the nuclear genome of *N. vitripennis *(9,254 contigs). The annotation of the nuclear genome of *A. mellifera *was used as a reference when searching for orthologs (see Methods, step [a.III]), and corresponding sequences of this species were added during this step. After the first processing steps [a.I/b.I to II], including a search for orthologs, a sequence check with *checking_seq*, filtering for longest sequence per species and gene, and excluding groups of orthologs with fewer than four species, the data set included a total of 13,573 sequences from 4,536 species and 375 genes. Step [III], the exclusion of species with only one sequence in the data set, led to the exclusion of 1,074 species and subsequently of 68 groups of orthologs. Accordingly, sequences of 3,462 species in 307 groups of orthologs were aligned in step [IV]. The selection of the first and second maximum cliques of species with an overlap of at least 100 nucleotides or amino acids [steps IX and X] and the subsequent tests for compositional heterogeneity [steps XI and XII] led to the exclusion of 669 species and reduced the data set to 2,793 species. The pruning of species-rich genera to 15 species led to the exclusion of another 549 species [step XIV]. Pruned genera were *Camponotus, Cardiocondyla, Dorylus, Lasius, Myrmecocystus, Pheidole, Pogonomyrmex, Polyrhachis, Pseudomyrmex *(Formicidae), *Bombus, Diadasia, Euglossa, Xylocopa *(Apidae), *Colletes, Hylaeus *(Colletidae), *Aleiodes, Cotesia *(Braconidae), *Ceratosolen *(Agaonidae), *Andricus *(Cynipidae), *Neodiprion *(Diprionidae), *Pontania *(Tenthredinidae), *Megastigmus *(Torymidae) and *Polistes *(Vespidae).

**Figure 2:**
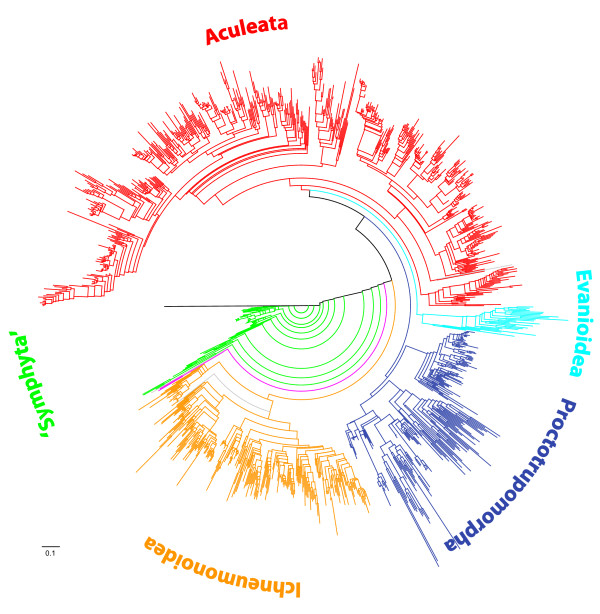
**Simplified phylogenetic tree of Hymenoptera inferred from GenBank sequences (tree 1 obtained from subset 1)**. The tree includes 1,142 species. The applied color code indicates major lineages.

**Figure 3:**
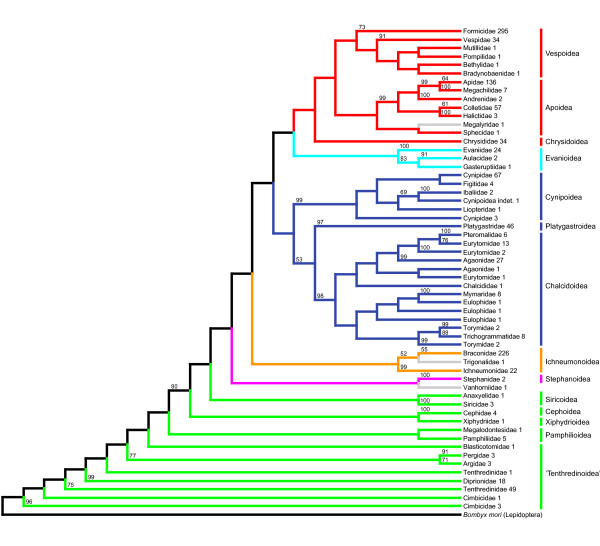
**Phylogenetic tree of Hymenoptera inferred from GenBank sequences (tree 1), reduced to family level**. Numbers that follow the family names indicate the number of analyzed species. Numbers above branches indicate bootstrap support values (%). Values < 50% are omitted. The applied color code corresponds to that of Figure 2. Single species whose position in the inferred phylogenetic tree we consider erroneous are shown in gray.

After selecting the largest group of species that overlap in at least one group of orthologs [step XV], the final concatenated data set (subset 1) included 1,146 species (46 families), 222 groups of orthologs, 3,951 sequences and 88,626 aligned sites. Data coverage in subset 1 (number of sequences ÷ number of groups of orthologs × number of species) was 1.55%. Tree reconstruction and 560 rapid bootstrap replicates took 8.3 days. Tree 1 obtained from subset 1 is shown in Figures 2 and 3 and Additional file 5.

**Figure 4:**
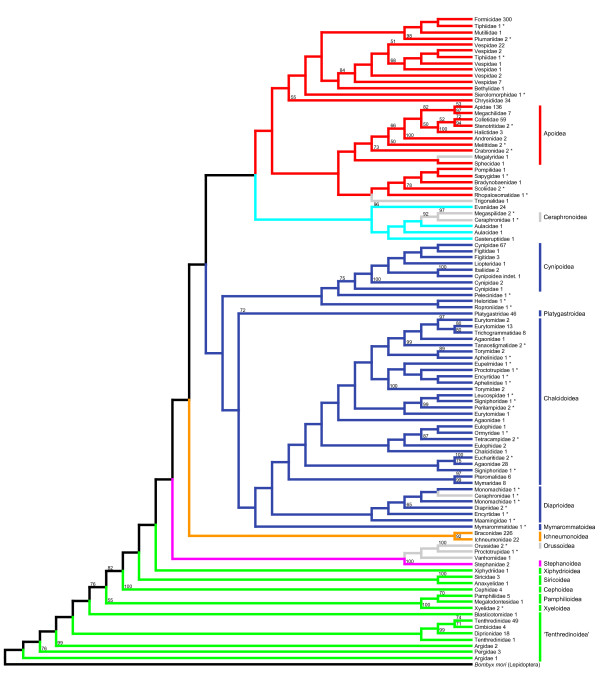
**Phylogenetic tree of Hymenoptera inferred from GenBank sequences (tree 2 obtained from subset 2), reduced to family level**. In this tree, species that were excluded by our pipeline in the course of generating subset 1 are reincluded. These taxa are marked with asterisks. The meaning of numbers and the applied color code correspond to those in Figure 3.

Subset 2 included an additional 115 sequences of 51 species from 31 families. Overall, the concatenated subset 2 consisted of 1,207 species (77 families), 222 groups of orthologs, 4,005 sequences and 88,807 aligned sites. The number of species is > 1,146 plus 51 due to repeated tests for compositional heterogeneity with slightly different results. (Both subsets are available at http://www.zfmk.de/web/Forschung/Molekularlabor/Datenstze/index.en.html). Data coverage (number of sequences ÷ number of groups of orthologs × number of species) in subset 2 was 1.49%. Tree reconstruction and 512 rapid bootstrap replicates took 8.9 days. Tree 2 obtained from subset 2 is shown in Figure 4 and Additional file 6. All species and all groups of orthologs included in subsets 1 and 2 are listed in Additional files 7, 8, 9 and 10.

## Discussion

The aim of the present investigation was to develop a bioinformatics pipeline for retrieving, processing, filtering, editing and analyzing large amounts of sequence data from GenBank in a phylogenetic context. Instead of using supertree approaches to explore existing data (see, for example, [19,49]), we relied on a direct reanalysis of the sequence data. Smith *et al*. [50] presented an alternative approach that they called a "mega-phylogeny approach", which also directly uses sequence data. It includes an *a priori *selection of gene regions of interest and an *a priori *separation of sequences into alleged monophyla with the aims of reducing the size of the supermatrix and improving alignment quality. A number of taxon-specific studies have also made use of GenBank sequence data, but those studies focused on specific genes (see, for example, [51,52]). We intended to avoid *a priori *decisions. In our pipeline, we suggest solutions for almost any obstacle that may appear along the way from sequence retrieval to tree reconstruction under the ML optimality criterion. In various regards, our approach is an extension and improvement of earlier efforts [2,4]. It offers an extended degree of automation in steps such as downloading from GenBank, sorting of sequences and translating and backtranslating sequences [steps I, b.II, b.V and VI] (Figure 1). Also, our approach includes improved quality management, such as by automatically checking the GenBank sequences for strand polarity and annotation, by masking problematic alignment regions and by handling compositional heterogeneity [steps b.III, VII and XI] (Figure 1). Our data selection steps [for example, steps III, IX and XV] (Figure 1) guarantee standardized levels of the density of the data set and of sequence overlap between included species. By choosing a minimum sequence overlap of 100 positions, we attempted to find a reasonable compromise between sequence overlap and number of species in the analysis. A larger overlap would have led to a significant decrease of the number of species in our phylogenetic tree. Furthermore, the present study is an update in terms of tree reconstruction facilities. We have, for the first time, applied a ML algorithm to such a large amount of GenBank data [step XVII] (Figure 1). Our approach is more general and independent of the taxonomic group. Finally, our bioinformatics solution is transparent and user-friendly. We provide all new scripts with respective comments and detailed manuals as part of this publication so that the pipeline is ready for use by anybody interested. In the following paragraphs, we discuss the results of our exemplary pipeline run with Hymenoptera data.

### Data set and analysis

One of the main characteristics of data sets when combining sequence data from independently conducted investigations is data scarcity; that is, the lack of data overlap. Data distribution in supermatrices is unbalanced, and, as a consequence, there is a huge amount of missing data. However, data sets do not necessarily have to be complete to provide phylogenetic information. In fact, there is evidence that even with very low coverage, reliable phylogenetic estimates can be obtained (see, for example, [53]). The sheer proportion of missing data is not decisive as long as the number of characters scored is sufficient to correctly place the taxa in the tree [54]. Accordingly, we tried to cope with the problem of data scarcity by ensuring a minimum sequence overlap between taxa and a standardized data set density [steps III, IX, XIII, XIV and XV] (Figure 1). Still, our Hymenoptera data matrix is very large and exhibits very low coverage (1.5%). This is a direct consequence of the characteristics of the original sequence information present in GenBank. A large number of species for which only few sequences are available contrasts with a small number of species for which the transcriptome, the mitochondrial genome or even the entire nuclear genome have been sequenced. By combining all of these data in a single analysis, this data set will inevitably become large and unbalanced and will suffer from low overlap between taxa. Irrespective of the fact that sequencing is getting cheaper and faster and that phylogenomic data will rapidly increase the size of data sets, the data characteristics described herein are still expected to prevail in the near future. The challenge is to find optimal subsets for phylogenetic analysis in order to explore available information and to subsequently identify and fill the most severe gaps via target-specific sequencing. Accordingly, one of the goals of our approach has been to identify unstable nodes and to suggest future foci of molecular phylogenetic studies, in Hymenoptera, for an effective, economical and time-saving process.

For tree reconstruction, we performed supermatrix ML analyses. To the best of our knowledge, this is the largest set of eukaryotic real data studied using ML analysis. Past studies that utilized very large data sets applied supertrees or parsimony analyses. For example, McMahon and Sanderson [2] and Thomson and Shaffer [4] applied maximum parsimony analyses with supermatrices in their pipelines, but stated that they based this decision mainly on speed and computational capacity. However, with the latest program version of RAxML implementing partitioned analysis, rapid bootstrap functions, and the ability of parallel analyses, even very large data sets, can be analyzed in a reasonable amount of time. In the next few years, systematic biologists' access to multicore computers will get easier and broader, and high-performance computing (HPC) will become routine. At the moment, subsets should be constrained in size to allow ML analysis. During our work, we set an approximate maximum of 1,500 taxa and 100,000 sites. Phylogenetic analyses of subsets of this size take a maximum of two weeks on a fully parallelized HPC unit such as the one that we used. Unless one wants to analyze data sets that are significantly larger than ours, there is no computational or speed argument left to perform supertree or parsimony methods in favor of ML analyses. Accordingly, our approach was designed to prepare data for ML analysis. However, if a user wants to apply other algorithms for tree reconstruction (for example, maximum parsimony) or to adjust parameters (for example, to seek an extension of exploration of tree space or a comparison between inferred trees), the supermatrix produced by our pipeline can be used just as well (after step XVI) (Figure 1).

### The phylogeny of Hymenoptera

We have restricted our results and discussion to (1) new contributions to the phylogeny of major lineages within Apocrita and to the monophyly and phylogeny of Proctotrupomorpha, (2) the recovery of some noncontroversial relationships and (3) the diagnosis of persistent problems and possible solutions. Phylogenetic relations within Hymenoptera are far too numerous and complex to be exhaustively discussed. The complete trees in Additional files 5 and 6 can be consulted for lower systematic level relationships.

In the following subsections, we repeatedly refer to single species as "misplaced". This means that their position as inferred in our trees clearly contradicts previous results from taxonomic as well as morphological and molecular phylogenetic studies. Accordingly, the phylogenetic positions of these taxa were considered artefacts and were excluded from discussion of topologies.

### Major lineages within Apocrita

Within Apocrita, our analysis suggests a topology of Stephanoidea + (Ichneumonoidea + (Proctotrupomorpha + (Evanioidea + Aculeata))) (with misplacement of a single Vanhorniidae as sister to Stephanoidea being ignored) (Figure 3). Stephanoidea was inferred to be sister group to all other Apocrita in the morphological analyses of Vilhelmsen *et al*. [17]. Our analysis gives additional support for this relationship. The Ichneumonoidea are monophyletic in our trees. (Misplacement of a single Trigonalidae as sister to Braconidae is ignored.) Ichneumonoidea has been suggested as sister group to Aculeata by Rasnitsyn [13], a relationship that found only moderate support from Vilhelmsen *et al*. [17] and was not retrieved by most recent analyses (see, for example, [16,21,24,55,56]). Our trees corroborate the results of most analyses cited above and suggest a rejection of the clade Aculeata + Ichneumonoidea. Instead, we found Evanioidea to be sister group to Aculeata in our trees. A sister group relationship of Evanioidea and Aculeata has been suggested only by the combined morphological and molecular analysis by Sharkey *et al*. [57], and there are currently no convincing morphological synapomorphies that would support this clade. However, despite low branch support, we consider it quite possible that the Evanioidea are the long-sought sister group to the Aculeata and suggest further investigation of this particular clade. Rasnitsyn [13] introduced the supertaxon Evaniomorpha, which includes Evanioidea, Ceraphronoidea, Megalyroidea, Trigonaloidea and Stephanoidea. We argue against the monophyly of Evaniomorpha, as our data support Stephanoidea as sister taxon of the remaining Apocrita (corroborating Vilhelmsen *et al*. [17]). We cannot provide substantial information on the position of the superfamilies Ceraphronoidea, Megalyroidea and Trigonaloidea, because their representatives are either included solely in the extended, possibly less reliable tree 2 (Ceraphronoidea) or obviously misplaced (Megalyroidea and Trigonaloidea).

### Proctotrupomorpha

In our analyses, Proctotrupomorpha *s.l*. (that is, sensu Rasnitsyn 1988 [13]) was retrieved when again ignoring a few misplaced taxa. In tree 1, Proctotrupomorpha comprises Chalcidoidea, Platygastroidea and Cynipoidea (all of which are monophyletic, forming Cynipoidea + (Platygastroidea + Chalcidoidea)). In tree 2, more representatives of Proctotrupomorpha *s.l*. are present, and the inferred topology suggests the following relationships: Cynipoidea + (Platygastroidea + (Mymarommatoidea + (Diaprioidea + Chalcidoidea))). This contradicts the often proposed sister group relationship between Mymarommatoidea and Chalcidoidea (see, for example, [24,57,58]; but see the ambiguity in [17]). A sister group relationship between Diaprioidea and Chalcidoidea was retrieved in the molecular analyses of Castro and Dowton [56], but their taxon sampling lacked Mymarommatoidea, and was retrieved by Heraty *et al*. [21]. Our study is one of the first to include Mymarommatoidea in a molecular phylogenetic analysis, but the position of Mymarommatoidea in our analysis is not well supported and the group is represented only in the less reliable tree 2. A position of Chalcidoidea outside Proctotrupomorpha was recently proposed by Sharanowski *et al*. [20] based on the analysis of 24 putative orthologous genes (derived from ESTs) from a small number of taxa. We regard this position as unlikely based on our own results and those of previous molecular studies that provided respective parts of our data set [16,21,56]. The most recent morphological or combined morphological and molecular analyses also contradict an origin of Chalcidoidea outside Proctotrupomorpha [17,57].

### Recovery of noncontroversial relationships

We evaluated the reliability of the inferred phylogenetic trees by the recovery of phylogenetic relationships that are largely considered noncontroversial. We found positive indications in tree 1. Specifically, our results are consistent with the generally accepted paraphyly of "Symphyta" (see, for example, [24]) and with the generally accepted monophyly of Apocrita and Aculeata (see, for example, [24,28]) (with misplacement of one Megalyridae within Aculeata being ignored). Also, we retrieved the noncontroversially monophyletic superfamilies Apoidea, Chalcidoidea, Cynipoidea, Evanioidea, Ichneumonoidea and Siricoidea. However, some crucial taxa were not represented in tree 1: Xyelidae and Orussidae. If we add them to the data set to infer tree 2, they are misplaced. The Xyelidae are found as a sister group to Pamphilioidea (Figure 4). This position is not very likely, as the sister group relationship of Xyelidae and the remaining Hymenoptera is well supported [25-27]. The Orussidae, which have a key position within Hymenoptera evolution as sister group of Apocrita, are placed at the base of Apocrita along with some Proctotrupoidea taxa (Figure 4). However, the clade Orussidae + Apocrita is well established and supported by morphological and molecular data (see, for example, [13,17,18,57]). This demonstrates the necessity of sequence overlap definitions and shows that the positions of reincluded taxa (indicated by asterisks in Figure 4 and Additional file 6) have to be discussed with caution. The backbone of the tree, with its major splits, however, remains largely unaffected by adding taxa that do not fulfill our overlap criteria.

### Diagnosis of persistent problems and possible solutions

With the aid of our trees, we identified several persistent problems in the Hymenoptera tree. While the available sequence data already cover all major lineages of Hymenoptera, they are unequally distributed and there is poor overlap among taxa. This contradiction between taxonomic breadth and genomic depth in the data of Hymenoptera is in accordance with the conclusions of Sanderson [59] in his evaluation of the phylogenetic signal in Eukaryota. The large amount of missing data and the low taxonomic overlap between mitochondrial and nuclear data in our sets call for a solution. To get more independent markers and to close the taxonomic gap between mitochondrial and nuclear data, we suggest EST studies (nuclear genes) for taxa with completely sequenced mitochondrial genomes and sequencing of mitochondrial genomes of those taxa for which we already have a large number of nuclear sequence data available.

An obvious problem for solving higher-level relationships within Hymenoptera is the underrepresentation of the small superfamilies Megalyroidea, Trigonaloidea, Ceraphronoidea and Mymarommatoidea. Another highly problematic issue is those families of Proctotrupoidea that we currently cannot map on the phylogenetic tree. Any additional data regarding these taxa in terms of species and genes will be of great value.

As extensive EST studies are still expensive, we also recommend target-specific amplification of nuclear coding genes. With the prospect of new primer design tools (J. Borner, C. Pick, T. Burmester, unpublished data), amplification and sequencing of a data set of, for example, 22 taxa (all superfamilies) and 50 nuclear coding genes can be accomplished in a reasonable amount of time and at reasonable cost. Taxon sampling should again be based on taxa with completely sequenced mitochondrial genomes.

## Conclusions

Exemplarily for Hymenoptera, we have demonstrated that the tree reconstructed from our pipeline output can make a substantial contribution to the phylogeny of the taxon and that comprehensive results can complement the discrete inferences from the single studies that have produced the data that were reanalyzed. Inspired by McMahon and Sanderson [2] and Sanderson *et al*. [3], we found an adequate approach to analyze all currently available molecular data in a single phylogenetic study in a standardized and efficient way. The impossible child of the scientific community, a sequence data monster, can be tamed. Every systematic biologist, even without advanced programming and bioinformatics skills, is given the capability to produce a tree of his taxon of interest. Our approach offers the possibility of relatively simple and reliable monitoring of new data and tree robustness, that is, the possibility to keep track of the phylogenetic signal in a taxonomic group. This also enables researchers to monitor how phylogenetic trees change over time with an increase of data size and density. This might promote a better understanding of more theoretical issues related to the analyses of molecular data, such as the information content of genes or the suitability and selection of genes to answer phylogenetic questions. Our approach therefore has great potential to meet the challenges of the phylogenomic era, to improve our ideas on phylogenetic affinities and to contribute to a better understanding of the evolution of organisms.

## Authors' contributions

BMi and RSP conceived of the study. BMe, BMi and RSP designed the study. RSP coordinated the study. BMe, JB, KM and RSP carried out the analyses. BMe wrote the major part of the bioinformatics tools, and JB and BMi wrote minor parts of the bioinformatics tools. BMe, JB and RSP wrote the manuals with comments and revisions from KM. BMe, BMi, ON and RSP wrote the manuscript. JB, KM, KS and LK provided comments on and made revisions to the manuscript. All authors read and approved the final manuscript.

## Supplementary Material

Additional file 1 **Software tools and manuals**. All newly developed software tools and corresponding manuals. Click here for file

Additional file 2 **gi numbers of sequences from GenBank used in our pipeline**. List of the GenBank gi numbers of all Hymenoptera sequences that were initially inputted in our pipeline run. Click here for file

Additional file 3 **On maximum cliques**. A short introduction to maximum cliques and how we used them in our analysis. Click here for file

Additional file 4 **Character partitions of subset 1 and 2**. The character partitions of the two subsets that were used in the phylogenetic analyses (subset 1 and subset 2). Click here for file

Additional file 5 **Tree 1, complete**. Phylogenetic tree of Hymenoptera inferred from GenBank sequences (tree 1). Numbers on branches indicate bootstrap support values (%). The applied color code corresponds to that of Figures 2 and 3. Single species whose position in the inferred phylogenetic tree we consider erroneous are shown in gray. Click here for file

Additional file 6 **Tree 2, complete**. Phylogenetic tree of Hymenoptera inferred from GenBank sequences (tree 2). In this tree, species that were excluded by our pipeline in the course of generating subset 1 are reincluded. These taxa are marked with asterisks. The meaning of numbers and the applied color code correspond to those in Additional file 5. Click here for file

Additional file 7 **Species included in subset 1**. All species included in subset 1, sorted by family. Click here for file

Additional file 8 **Groups of orthologs included in subset 1**. All groups of orthologs included in subset 1, plus coverage of each group. Click here for file

Additional file 9 **Species included in subset 2**. All species included in subset 2, sorted by family. Click here for file

Additional file 10 **Groups of orthologs included in subset 2**. All groups of orthologs included in subset 2, plus coverage of each group. Click here for file
