# Supplementary material for: The taming of an impossible child: a standardized all-in approach to the phylogeny of Hymenoptera using public database sequences
Source: BMC Biol. 2011 Aug 18;9:55. doi: 10.1186/1741-7007-9-55 (PMC3173391; doi:10.1186/1741-7007-9-55)
Supplement: Additional file 3 — On maximum cliques. A short introduction to maximum cliques and how we used them in our analysis. [file 1741-7007-9-55-S3.PDF]

On maximum cliques

Used in pipeline steps [IX, X]:

Select maximum clique of seqs with ≥ 100nt or ≥ 100aa overlap

----

In graph theory, an undirected graph consists of vertices and edges, where an edge represents the connection between two vertices. A clique in a graph G is a complete subgraph of G. Thus, each vertex of the subgraph is connected to all other vertices of the subgraph. If a clique cannot be extended by any further vertices of G, it is called a maximal clique (Figure 1). The maximum clique of G is defined as the largest clique of G.

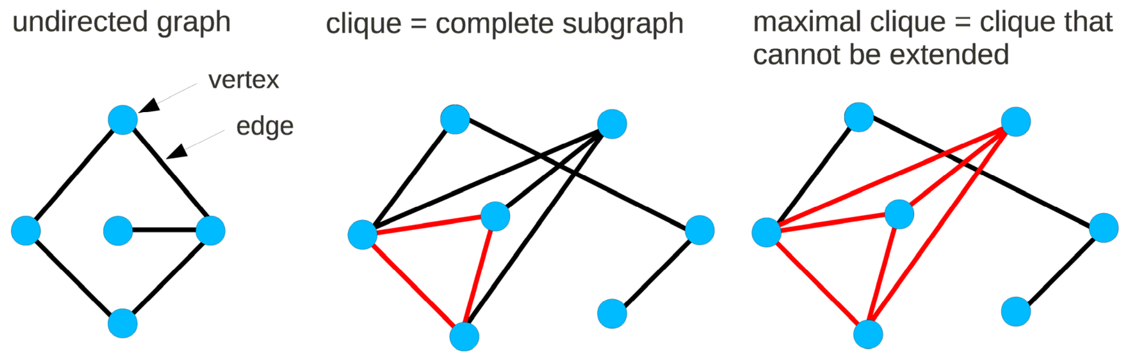

Figure 1. Cliques and maximal cliques in graph theory.

Our aim was to identify the highest number of species, where each species pair ij has an overlap of ≥ 100 nucleotides/aminoacids. Therefore, we checked the overlap of each pair ij, which results in a lower triangular presence absence matrix M, with m_ij = 1 if species i and j have an overlap of ≥ 100 nucleotides/amnioacids, otherwise m_ij = 0. M can be transformed into a graph G(M), where each species i is represented by a vertex v_i. If m_ij = 1, corresponding vertices v_i and v_j are connected by an edge. Thus, the largest clique in G(M) identifies the largest group of species, where each species pair ij has an overlap of ≥

100 nucleotides/aminoacids. We used the program 'cliquer' (Niskanen and Östergård, 2003) to identify maximum cliques in a given graph.

Reference:

Niskanen S, Östergård PRJ: **Cliquer User's Guide, Version 1.0.** Communications Laboratory, Helsinki University of Technology, Espoo, Finland, Tech. Rep. T48, 2003

cliquer can be downloaded at: http://users.tkk.fi/pat/cliquer.html
