# Supplementary material for: The taming of an impossible child: a standardized all-in approach to the phylogeny of Hymenoptera using public database sequences
Source: BMC Biol. 2011 Aug 18;9:55. doi: 10.1186/1741-7007-9-55 (PMC3173391; doi:10.1186/1741-7007-9-55)
Supplement: Additional file 4 — Character partitions of subset 1 and 2. The character partitions of the two subsets that were used in the phylogenetic analyses (subset 1 and subset 2). [file 1741-7007-9-55-S4.PDF]

Character partitions of subset 1 and 2

Partitions subset 1

COX3, first maximum clique = 1-528
COX2, first maximum clique = 529-916
16S, second maximum clique = 917-1413
ND2, first maximum clique = 1414-2103
12S, second maximum clique = 2104-2543
ND1, second maximum clique = 2544-2997
ITS2, first maximum clique = 2998-4099
COX2, second maximum clique = 4100-4487
ND6, first maximum clique = 4488-4873
ITS2, second group of taxa with compositional homogeneity = 4874-5975
tRNAs concatenated, first maximum clique = 5976-6522
18S, first maximum clique = 6523-7333
28S, first maximum clique = 7334-8067
ND3, first maximum clique = 8068-8301
COX1, first maximum clique = 8302-9267
ND5, first maximum clique = 9268-10403
18S, second group of taxa with compositional homogeneity = 10404-11214
ATP8, first maximum clique = 11215-11322
ATP6, second maximum clique = 11323-11778
12S, first maximum clique = 11779-12218
COX2, second group of taxa with compositional homogeneity = 12219-12606
16S, second group of taxa with compositional homogeneity = 12607-13103
ND4, first maximum clique = 13104-14001
ATP6, first maximum clique = 14002-14457
CytoB, first maximum clique = 14458-14839
28S, second group of taxa with compositional homogeneity = 14840-15573
28S, second maximum clique = 15574-16307
16S, first maximum clique = 16308-16804
ND4L, first maximum clique = 16805-17000
ND1, first maximum clique = 17001-17454
ATP8, second group of taxa with compositional homogeneity = 17455-17562
nuclear protein-coding seqs = 17563-88626

Partitions subset 2

COX2, first maximum clique = 1-388

18S, first maximum clique = 389-1199

ND2, first maximum clique = 1200-1889

CytoB, first maximum clique = 1890-2271

COX2, second maximum clique = 2272-2659

ND6, first maximum clique = 2660-3045

ITS2, second group of taxa with compositional homogeneity = 3046-4147

tRNAs concatenated, first maximum clique = 4148-4694

12S, second maximum clique = 4695-5134

COX1, first maximum clique = 5135-6100

ND5, first maximum clique = 6101-7236

18S, second group of taxa with compositional homogeneity = 7237-8047

ND3, first maximum clique = 8048-8281

16S, first maximum clique = 8282-8778

16S, second maximum clique = 8779-9275

ATP8, first maximum clique = 9276-9383

COX3, first maximum clique = 9384-9911

12S, first maximum clique = 9912-10351

COX2, second group of taxa with compositional homogeneity = 10352-10739

16S, second group of taxa with compositional homogeneity = 10740-11236

ND4, first maximum clique, 11237-12134

ATP6, first maximum clique = 12135-12590

ND1, second maximum clique = 12591-13044

28S, second group of taxa with compositional homogeneity = 13045-13778

ATP6, second maximum clique = 13779-14234

28S, second maximum clique = 14235-14968

ND4L, first maximum clique = 14969-15164

ND1, first maximum clique = 15165-15618

ITS2, first maximum clique = 15619-16720

ATP8, second group of taxa with compositional homogeneity = 16721-16828

28S, first maximum clique = 16829-17743

nuclear protein-coding seqs = 17744-88807
