# Supplementary material for: The taming of an impossible child: a standardized all-in approach to the phylogeny of Hymenoptera using public database sequences
Source: BMC Biol. 2011 Aug 18;9:55. doi: 10.1186/1741-7007-9-55 (PMC3173391; doi:10.1186/1741-7007-9-55)

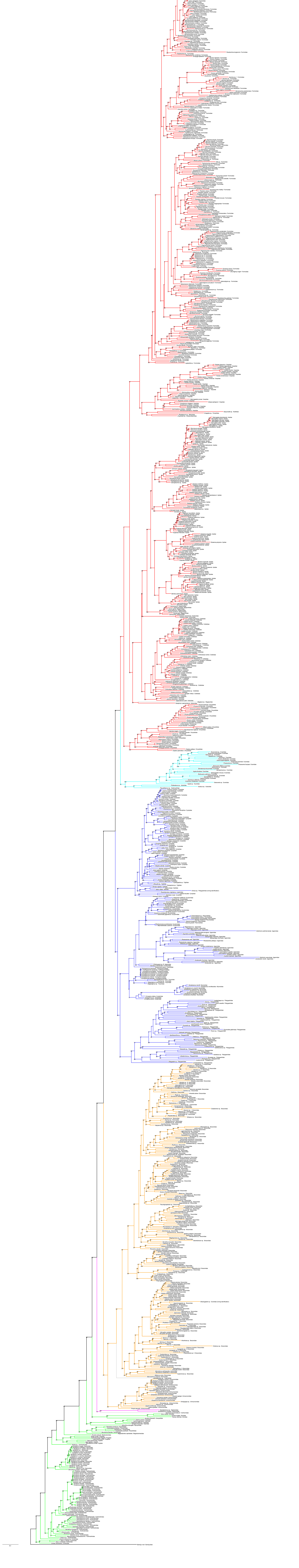

Supplement: Additional file 5 — Tree 1, complete. Phylogenetic tree of Hymenoptera inferred from GenBank sequences (tree 1). Numbers on branches indicate bootstrap support values (%). The applied color code corresponds to that of Figures 2 and 3. Single species whose position in the inferred phylogenetic tree we consider erroneous are shown in gray. [file 1741-7007-9-55-S5.PDF]
