# Supplementary material for: The taming of an impossible child: a standardized all-in approach to the phylogeny of Hymenoptera using public database sequences
Source: BMC Biol. 2011 Aug 18;9:55. doi: 10.1186/1741-7007-9-55 (PMC3173391; doi:10.1186/1741-7007-9-55)

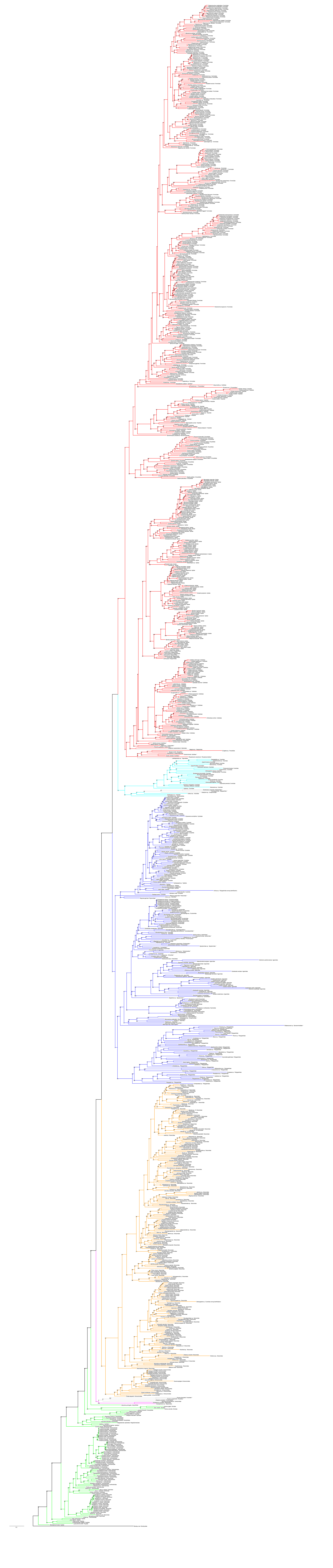

Supplement: Additional file 6 — Tree 2, complete. Phylogenetic tree of Hymenoptera inferred from GenBank sequences (tree 2). In this tree, species that were excluded by our pipeline in the course of generating subset 1 are reincluded. These taxa are marked with asterisks. The meaning of numbers and the applied color code correspond to those in Additional file 5. [file 1741-7007-9-55-S6.PDF]
